# Towards the Operationalization of Health Technology Sustainability Assessment and the Early Eco Design of the Internet of Medical Things

**DOI:** 10.3390/s25133839

**Published:** 2025-06-20

**Authors:** Ernesto Quisbert-Trujillo, Nicolas Vuillerme

**Affiliations:** 1 AGEIS, Université Grenoble Alpes, 38000 Grenoble, France; ernesto.quisbert@univ-grenoble-alpes.fr; 2 Institut Universitaire de France, 75231 Paris, France

**Keywords:** sustainability, digital health, Internet of Things, Health Technology Assessment, cost-benefit assessment, cost-effectiveness assessment, Life Cycle Assessment, eco design

## Abstract

An increasing number of scholars are raising concerns about the sustainability of digital health, calling for action to prevent its harmful effects on the environment. At this point, however, the comprehensive appraisal of emerging technology in the health sector remains theoretically challenging, and highly difficult to implement in practice and in ecological design. Indeed, background factors such as the rapid evolution of technology or effectiveness–efficiency tradeoffs complicate the task of distinguishing the benefits of digital health from its drawbacks, rendering early Health Technology Sustainability Assessment (HTSA) extremely complex. Within this context, the aim of this article is to draw attention to the pragmatism that should be adopted when anticipating the sustainability of technological innovation in the medical field, while simultaneously proposing an assessment framework grounded in a structural and conceptual dissection of the fundamental purpose of smart technologies and the Internet of Medical Things (IoMT). Building on this, we demonstrate how our framework can be strategically applied through a rapid back-of-the-envelope assessment of the economic and ecological balance when introducing IoMT prototypes for treating a specific condition, based on a preliminary simulation of a defined clinical outcome. In this manner, the article presents evidence that challenges two primary hypotheses, and also encourages reflection on the central role of information and its interpretation when addressing key barriers in the HTSA of digital health. Thereby, it contributes to advancing cost–benefit and cost-effectiveness evaluation tools that support eco design strategies and guide informed decision-making regarding the integration of sustainable IoMT systems into healthcare.

## 1. Introduction

Digital health offers substantial benefits to patients and the medical field, yet it also introduces several vulnerabilities that could compromise these gains. For example, it facilitates the diagnosis, treatment, and monitoring of health conditions [1], optimizes healthcare operations, reduces administrative burdens, and ultimately shifts focus toward patient care [2]. However, it can also give rise to a range of undesirable environmental effects throughout the life cycle of enabling technology, including global warming [3], resource depletion [4] and electronic waste [5]. How can the advantages and disadvantages of technology be effectively differentiated to enable a comprehensive and pragmatic assessment and ecological design of digital health innovations?

In the context of smart technologies and the Internet of Medical Things (IoMT), addressing this research question is of utmost importance. Recent findings raise concerns that the anticipated benefits of commercially available IoMT solutions (e.g., reduction in hospital service use [6] or personalized medical assistance and monitor health [7]) may be deeply undermined by the negative implications of the excessive production of sensor devices [8,9], their uncontrolled disposal [4,10,11] and their electricity consumption [4,12,13].

In general, Health Technology Assessment (HTA) is the most widely adopted and endorsed approach for evaluating the balance between the returns and tradeoffs of innovations in the medical field. In fact, according to the World Health Organization (WHO), HTA represents a systematic evaluation of the properties, effects, and/or impacts of health technologies, intended to inform decision-making [14].

Recently, certain digital health technologies have been integrated into HTA frameworks to support not only decision-makers but also policymakers, particularly through cost–benefit, cost-effectiveness, and cost–utility analyses (CBA, CEA, CUA). Within the literature, the interest in telemedicine is considerable and, although most studies address common dimensions such as outcomes, ethical considerations, efficiency, and efficacy, their specific emphases vary significantly.

In terms of clinical and patient outcomes, for instance, some authors [15,16,17,18] focus on assessing the influence of telemedicine on mortality, morbidity, adherence, and quality of life, while others [19] investigate patient satisfaction and technology usability. Additionally, there are researchers that delve into emotional factors that affect patients’ quality of life [20].

With respect to ethical aspects, Grigsby et al. [17] examined the impact of telemedicine on the cost of care, whereas Khoja et al. [21] explored issues related to technology access and its social implications. Furthermore, Ekeland et al. [15] and Kidholm et al. [16] addressed socio-cultural and legal dimensions, while other authors [22,23,24] focused on cost, acceptability, and affordability.

In the context of efficiency of telemedicine, Brear M. [25] evaluated technical performance, other researchers [15,16,18,21] investigated the use and allocation of resources, and Ohinmaa et al. [19] examined diagnostic quality. Regarding efficacy, Ohinmaa et al. [19] addressed service quality and reliability, Khoja et al. [21] considered the appropriateness of technology, Ekeland et al. [15] and Kidholm et al. [16] explored technological maturity, Dechant et al. [23] assessed accuracy, and Brown et al. [26] and Shaw N.T. [27] focused on training in the use of digital health technologies.

On the other hand, the integration of environmental aspects into HTA and the examination of eHealth technologies through the lens of sustainability have drawn increasing scholarly attention in recent years. Regarding the green transition of HTA, Toolan et al. [3] identified four strategies to support HTA agencies in incorporating ecological criteria and conducting environmental impact assessments of healthcare innovations. These include reusing existing environmental data, analyzing environmental data separately from established economic evaluations, adopting or developing new methodologies, and focusing solely on environmental benefits (regardless of clinical outcomes).

A recent review by Iandolo et al. [28] underscored the lack of a holistic perspective in current impact estimation methodologies, especially in capturing the direct and indirect effects of innovation in healthcare. Williams et al. [29] show that some emerging tools address this gap by incorporating incremental carbon footprint cost ratios, or by integrating environmental impact into multi-criteria decision-making frameworks. However, as they point out, no existing method, framework, or tool is yet suitable for widespread use. Therefore, the further tailoring and adaptation of assessment methodologies appear to be inevitable [30].

Concerning the sustainability of eHealth technologies, Alajlan et al. [31] emphasized the importance of first establishing a clear definition of sustainability in healthcare before developing evaluation approaches. Their work highlights critical enabling factors—such as ICT infrastructure, hardware, and data/system quality—and calls for impact assessment methods that consider these alongside organizational, economic, social, and resource-related dimensions. In this context, Bobini et al. [32] analyzed the pivotal role of Life Cycle Assessment (LCA) in green HTA for telemedicine, concluding that accounting for both the environmental costs of equipment production and the environmental benefits of its use is essential. Along these lines, Brown, C. et al. [33] advocated for frugal innovation as a path to affordable, low-ecological-impact healthcare solutions.

While all this literature is undoubtedly valuable and enlightening, the specificities of smart technology remain largely unexplored, and the nuances of modern IoMT equipment have yet to be systematically integrated from both Health Technology Assessment and design perspectives.

Bridging this gap in knowledge is critically urgent, especially considering that, according to the WHO’s global strategy, the medical sector should already be improving physical and psychological well-being for all, everywhere, by accelerating the development and adoption of appropriate, accessible, affordable, scalable and sustainable person-centered digital solutions [34]. Clearly, they did not anticipate that integrating the sustainability assessment and eco design of smart technologies with a cost-effectiveness and comprehensive mindset is challenging, particularly in the case of IoMT systems and prototypes, which are conditioned to numerous issues such as the rapid evolution of hardware [35,36,37] or effectiveness–efficacy tradeoffs [38].

In this work, we strive to address this challenge, arguing that the integral Health Technology Assessment and eco design of sustainable smart technology and IoMT solutions should embrace a pragmatic approach primarily grounded in their capacities to sense, size and transform information.

### 1.1. Hypotheses

Sensing, sizing and transforming data are the most distinctive features of smart technology [39] and represent the primary functions of the Internet of Things, all designed to provide information to human and/or machines autonomously [40]. In the medical field, this suggests that “IoMT devices continuously sense and collect data, thereby generating *information* that can be subsequently analyzed and contextualized to implement medical treatments efficiently and effectively, and allocate healthcare resources optimally” (Hypothesis 1).

*Information*, on the other hand, can be defined as “the message from which a sustainable digital-based health service or paradigm can exist, and with which specific barriers for integral assessment and eco design can be surpassed” (Hypothesis 2).

### 1.2. Proposition

**Figure 1:**
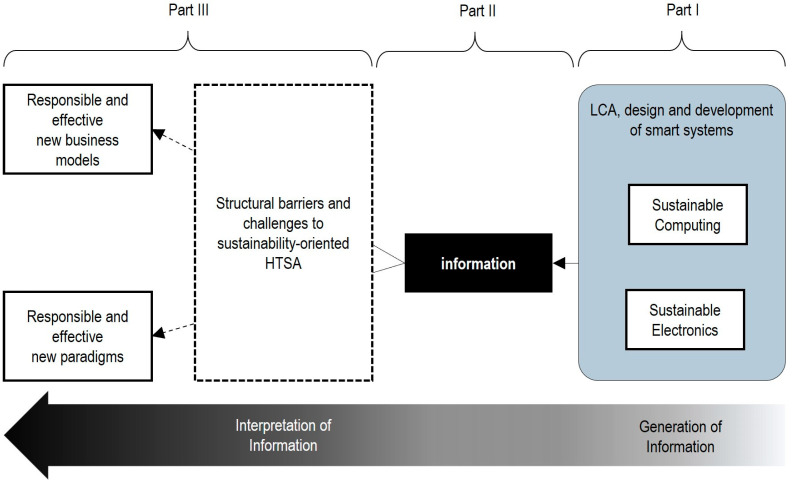
A novel Health Technology Sustainability Assessment (HTSA) framework for smart technologies and IoMT systems.

Building on our hypotheses and recognizing that smart technologies integrate advanced computing into smart systems (comprising local equipment and shared infrastructure [41]), we propose in Figure 1 a novel framework to pragmatically integrate sustainability and eco design into the Health Technology Assessment of smart technologies and IoMT systems.

This framework is grounded in information science theory and the Data–Information–Knowledge (DIK) hierarchy [42,43]. It consists of three parts, operating as follows: In part I, the Life Cycle Assessment, eco design and development of sustainable computing and electronic prototypes are addressed. In part II, only the sufficient data are collected or defined to generate the information for both the primary function and the evaluation of medical applications addressed by the system. Finally, Part III involves interpretation, which helps in addressing specific difficulties, barriers, challenges and dilemmas that hinder the integral cost-effectiveness analysis and development of new medical devices, business models and paradigms.

The aim of the next sections is to illustrate the use of this framework and test our hypotheses presented in Section 1.1.

With respect to the first hypothesis, this work will gather evidence to determine whether, based on specific information, a particular IoMT-based treatment can be made more effective while simultaneously reducing its implementation costs.

With respect to the second hypothesis, this work will gather evidence to determine whether, based on specific information, the potential cost–benefit balance of using IoMT devices in a medical treatment can be quantified in a practical and objective manner.

To this end, we project and contrast, through a systematic implementation of parts I, II and III of our proposed methodology, the economic and ecological cost–benefit sides of transforming a medical device into a “smart” medical device.

## 2. Materials and Methods

Our work is based on a secondary data analysis of a referential study [44] that evaluates the production cost and Social Cost of Carbon (SCC) emissions of reusable Soft Mist Inhalers (SMI) employed in three treatment patterns for Chronic Obstructive Pulmonary Diseases (COPD). Each treatment pattern follows an annual application cycle. Treatment pattern 1 involves the use of twelve single packs (each containing one reusable inhaler with one medication cartridge), treatment pattern 2 involves the combined use of three single packs and three triple packs (each containing 1 reusable inhaler with three cartridges) and treatment pattern 3 involves four triple packs. The reference study follows a budget impact model, in which the baseline population was estimated using market data representative of the current usage of the three brands included in the model in Germany: Spiriva^®^ (57%), Spiolto^®^ (42%), and Striverdi^®^ (1%) (all produced by Boehringer Ingelheim, a pharmaceutical company based in Ingelheim am Rhein, Germany).

From the estimations employed in this study, we isolate the part related to the reusable inhalers that administer Tiotropium bromide, namely, Spiriva cartridges—given their better documentation and traceability—and propose “smart” prototypes of them. Appendix A offers a more detailed account of the reference study, along with our preliminary preparation of the original data.

With the smart inhaler prototypes, we tackle poor inhaler technique (which is recognized as a core challenge in COPD treatment [45]) and, via our framework, we address the challenge of projecting the economic and environmental costs and benefits associated with improving this technique, based on a simulation-based, back-of-the-envelope analysis of drug delivery. Concretely, we estimate the economic and environmental cost of additional technology (part I of our proposed framework), establish the essential data and information addressed by the smart inhaler prototypes (part II) and, from that, evaluate the benefits (part III).

### 2.1. Economic and Environmental Cost of the Additional Technology (Part I)

Initially, the smart inhaler prototypes integrate an electronic module powered by a coin cell battery. Based on the core requirements related to tracking the inhalation profile of COPD patients, we conceptualize a feature-rich, a high-performance and a lightweight version of the electronic module in draft form (hereafter referred to as versions A, B and C, respectively). Then, we estimate individually their costs of production, and model and calculate their global warming impacts using Ecoinvent (3.10 edition). Finally, we project separately the economic cost and the SCC (By assuming an environmental charge of EUR 40 per ton of carbon dioxide (CO_2_), consistent with the baseline scenario of the reference study) of all smart inhaler prototypes used in each treatment pattern with Spiriva, designed according to the three proposed versions. Appendix A offers a more detailed account of the initial considerations underlying the implementation of this final step.

### 2.2. Essential Data and Information (Part II)

The smart inhaler prototypes are equipped with microelectromechanical system (MEMS) sensors detecting pressure drop (${∆}_{p}$) by inhalation airflow. As such, we model the inspiration flow rate (Q) of a COPD patient according to Equation (1), shown below.

(1) $$Q=\frac{\sqrt{{∆}_{p}}}{R}$$

Here, R corresponds to the device’s resistance (assumed to be 1 for the sake of simplicity).

**Figure 2:**
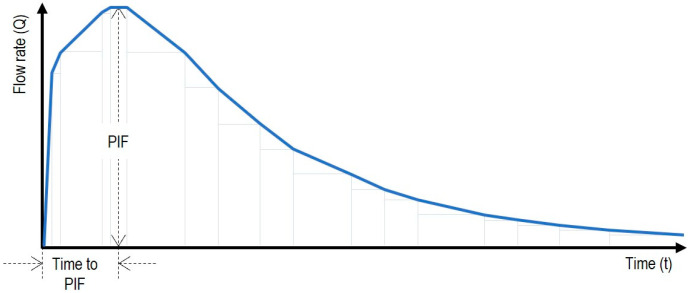
Simplified inhalation profile of a COPD patient showing his or her PIF and time-to-PIF.

Thus, when patients inhale, the pressure drop generated across the smart inhaler prototypes is positively correlated with the instantaneous inspiration flow rate achieved at any time before and after their “Peak Inspiratory Flow” (PIF) (Figure 2).

In this sense, a sensed pressure drop (${∆}_{p_{i}}$) constitutes the essential data input for deriving the inspiration flow rate ($Q_{i}$) at a given moment ($t_{i}$) before and after PIF. Meanwhile, the cumulative sum of the decomposed n geometric areas ($A_{n}$) under the curve in Figure 2 provides the information that approximates the inhalation volume (inhV) a patient can achieve when using a smart inhaler prototype.

### 2.3. Interpretation of the Information and Evaluation of Benefits (Part III)

#### 2.3.1. Interpretation of the Information

**Figure 3:**
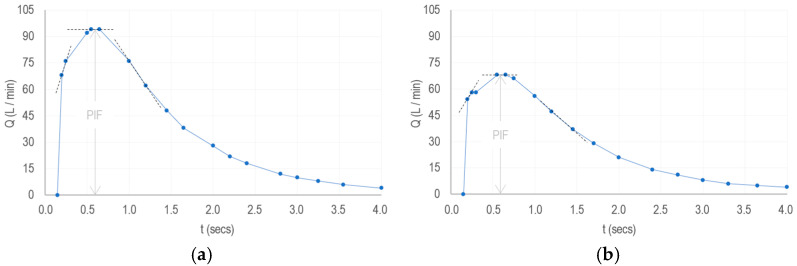
Simplified referential inhalation profiles (condensed into 18 data points from [46]). (**a**) Moderate COPD. (**b**) Severe COPD.

From the literature provided by the manufacturer of the reusable inhalers [46], we simplify the construction of the referential inhalation profiles of patients with moderate and severe COPD prescribed Spiriva (Figure 3).

From this, we can derive the following interpretations:

- The pressure drop measure ${∆}_{p_{i}}$ generated by a patient is proportional to the slope $m_{i}$ calculated between the data points collected from $t_{i-1}$ to $t_{i}$ (refer to the dashed lines in Figure 3a,b for illustration);
- The steeper the slopes before PIF and the less steep they are after, the greater the volume of medication inhaled and the more effectively the correct inhalation technique is acquired;
- The greater the volume of medication inhaled, the greater the drug delivery in the lungs (disregarding oropharyngeal deposition for the sake of simplicity);
- The greater the drug delivery, the less medication is needed in cartridges;
- The less medication content needed in cartridges, the lower the environmental and economic costs associated with their production.

#### 2.3.2. Evaluation of Benefits

Building on the above, the environmental and economic benefits are henceforth defined here as the environmental and economic savings associated with the use of reduced-content Spiriva cartridges in a sample of patients who adopted the proper inhalation technique over a specific time frame.

As for the proper technique, the manufacturer of the reusable inhalers recommends a slow, deep breath through their inhalers to ensure effective use [47]. When these basic instructions were applied in earlier investigations [48], in vitro experiments [46] yielded Spiriva delivery rates of approximately 59.2% in moderate COPD and 67.4% in severe COPD patients, although with variability levels of ±4.9% and ±4.6%, respectively.

Based on this, we proceed to (1) simulate the increase in the annual delivery rates of Spiriva achieved by COPD patients, who were assumed to all be assisted by the smart inhaler prototypes to correct their inhalation techniques over a training period of five and four years (from 2019 to 2023 and from 2020 to 2023, consistent with the reference study); and to (2) project the environmental and economic savings over the next five years (from 2024 to 2028), derived from the use of reduced-content Spiriva cartridges only by patients who have learned the proper inhalation technique.

##### Baseline Simulation of the Drug Delivery Rates

First, we adopt each of the referential inhalation profiles reported by Ciciliani, A. M., et al. [46] (refer to Figure 3), as approximations of a “slow, deep breath” taken by a moderate or severe COPD patient (simplified into 18 data points), and proceed to simulate and compare consecutively the slope values of every segment, propagating the error from one slope to the next.

Second, we calculate the inhalation volume ($inhV_{sim}$) from the generated inspiration flow rates ($Q_{1}$, … $Q_{18}$) and the geometric areas formed in each segment.

Third, for each training year, we simulate a low drug delivery rate if the calculated $inhV_{sim}$ value is lower than the one calculated from the referential inhalations profiles (inhV), or a high drug delivery rate in the opposite case.

We adopt four Monte Carlo models, each with 104,500 runs, whereby a single run represents a patient in the training process. Consistent with the reference study, two models were developed for moderate and severe COPD patients who were assumed to be trained with the smart inhaler prototypes over a period of 5 years (from 2019 to 2023), and other two models for moderate and severe COPD patients who were assumed to be trained for only 4 years (from 2020 to 2023).

We adopt a conservative scenario, aligned with previous evidence and based on the following assumptions:

- Throughout the two consecutive inhalations required for the daily administration of Spiriva [49], a patient tries to correct his or her technique through the second inhalation only if the $inhV_{sim}$ value obtained in the first inhalation is lower than the reference inhV value;
- The annual slope values are normally distributed, with those calculated from the reference inhalation profiles considered as the averages (refer to Table S1 of the Supplementary Material);
- The slope values exhibit high-to-moderate variations before their PIF (as related research [50] reports high time variability prior to this point), and moderate-to-low variations thereafter (as the slopes rapidly approach zero afterward);
- The initial variations of slope values progressively decrease, but only if the correct inhalation technique is confirmed each year (i.e., when $inhV_{sim}$ ≥ inhV);
- The annual delivery rates of Spiriva are normally distributed, and left- and right-truncated at the mean values 59.2% and 67.4%, respectively, corresponding to the rates reported for patients with moderate and severe COPD [46];
- The drug delivery rates progress annually with low-to-moderate variations only if the correct inhalation technique is confirmed each year (achieving, at best, historical variations of +4.9% and +4.6% for moderate and severe COPD patients [46], respectively, in the final training year);
- Only patients who have consistently learned the correct inhalation technique over the course of the five or four training years are considered to have acquired the proper inhalation technique.

The Supplementary Material provides more details related to this section.

##### Estimation of the Economic and Environmental Cost and Savings

We project the economic and environmental costs and savings associated with introducing alternative Spiriva cartridges into the treatment of moderate and severe COPD with reusable and smart reusable prototypes over the next five post-training years (from 2024 to 2028), where their content reduction is proportional to the simulated increase in drug delivery rates.

For this, we add the economic and environmental costs of each of the three design versions of the electronic module and the economic and environmental costs of a reusable inhaler (including the standard-content cartridge); we assume an approximate environmental impact of 80 g CO_2_-eq per standard-content cartridge (according to Hänsel, M., et al. [51]), and we proceed under the following policies:

- Default policy. Prescribe the smart inhaler prototypes to all patients, but with reduced-content cartridges only for those that have learned the proper inhalation technique;
- Alternative policy. Prescribe the smart inhaler prototypes with standard-content cartridges only to patients that have not learned the proper inhalation technique, and regular reusable inhalers with reduced-content cartridges to the remaining patients (assuming that the latter have improved drug delivery and no longer require the smart inhaler prototypes to correct their inhalation technique).

We consider five policy configurations along with the three treatment patterns in our analysis—three configurations considering the three design versions of the electronic module and the default policy (SR1 including design version A of the electronic module, SR2 including design version B and SR3 including design version C), and two other configurations considering two design versions and the alternative policy (SR2M including design version B and SR3M including design version C).

**Figure 4:**
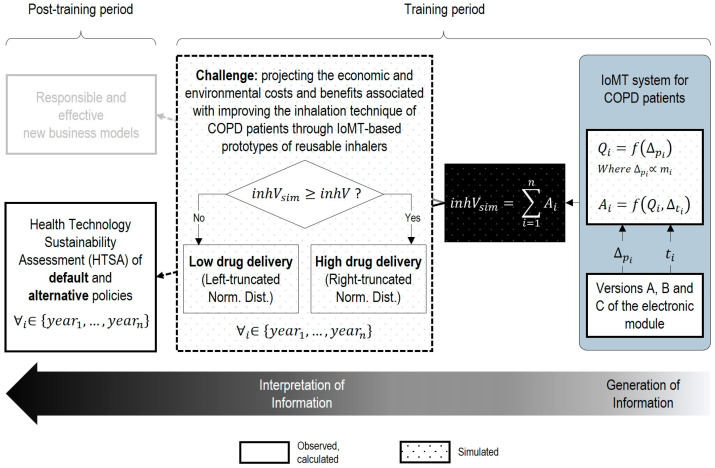
HTSA for the implementation of an IoMT system in inhaler-based COPD treatment.

In summary, Figure 4 synthesizes the most relevant aspects discussed thus far to operationalize each part of our proposed framework in the context of our case study.

## 3. Results

### 3.1. Economic and Environmental Cost of the Additional Technology

Table 1 and Table 2 provide, respectively, a summary of the main components of the three design versions of the electronic module, and a summary of their production costs and environmental impacts (more details can be found in Appendix B).

Accordingly, Table 3 below summarizes the economic and environmental costs of the smart inhaler prototypes in their three design versions, used by all patients over the course of a year, under the three treatment patterns with standard-content Spiriva cartridges.

### 3.2. Annual Increase in the Delivery Rates of Spiriva Gained During the Training Period

The findings derived from the four Monte Carlo models indicate that approximately 32% of the moderate and severe COPD patients are likely to adopt the proper inhalation technique after a five-year training period, whereas only 23% are likely to do so after four years. Table 4 below details the year-by-year average increase in delivery rates of Spiriva achieved by these patient groups.

### 3.3. Economic and Environmental Cost and Savings

#### 3.3.1. Annual Economic Costs and Savings

Table 5 below presents the annual cost of an alternative Spiriva cartridge during the post-training period after subtracting the drug content proportional to the increase in delivery rates reported in Table 4.

Accordingly, Table 6 below provides the average costs of the default policy (prescribe the smart inhaler prototypes to all patients, but with reduced-content cartridges only for those that have learned the correct inhalation technique) in its configurations SR1, SR2 and SR3.

Table 7 below presents the respective annual savings.

Notice that employing the design versions A, B, and C under the default policy results in identical savings across the three treatment patterns, as the increase in annual drug delivery rates is uniformly applied to all three.

On the other hand, the average costs and savings associated with the alternative policy (prescribe the smart inhaler prototypes with standard-content cartridge only to patients that have not learned the proper inhalation technique, and the regular reusable inhalers with reduced-content cartridge to remaining patients) are presented in Table 8 and Table 9 (for its SR2M and SR3M configurations, respectively).

Note that even with a training period limited to four years, cost savings in 2024 are achieved without reducing the content of Spiriva cartridges. This is because patients who had previously mastered the proper inhalation technique were provided later with standard reusable inhalers, which are less costly than their smart counterparts.

Moreover, the estimated cost savings derived from policy configuration SR3M exceed those of policy configuration SR2M, primarily because the initial investment required for the former is greater than that for the latter (while the savings derived from using reduced-content cartridges are identical in both cases). In this vein, the cost savings observed in treatment pattern 1 are greater than those in treatment pattern 2, which in turn exceed those in treatment pattern 3, as the annual investment per patient is substantially higher in the first case (twelve inhalers), while it is comparatively lower in the second and third cases (six and four inhalers, respectively).

#### 3.3.2. Annual Environmental Cost and Savings

Table 10 presents the annual cost of an alternative Spiriva cartridge after subtracting the drug content proportional to the increase in delivery rates reported in Table 4.

Based on this, Table 11 and Table 12 respectively present the average costs and annual savings of the policy configurations SR1, SR2 and SR3.

The savings derived from using the three design versions of the smart inhaler prototypes under the default policy are comparable across the three treatment patterns. However, the cost of policy configuration SR3 is at this point significantly reduced, primarily due to the lightweight design version C of the electronic module.

To conclude this section, Table 13 and Table 14 summarize the annual cost and savings associated with policy configurations SR2M and SR3M, mirroring the interpretations adopted from the economic counterpart.

## 4. Discussion

The literature somewhat related to the focus of this article does not reconcile HTA and the design of the enabling technology, and much less from a sustainability perspective. For example, the methodology proposed by Caulfield et al. [58] enables the identification and evaluation of suitable sensor devices for healthcare applications based on specific requirements (such as timeframe, objectives, and data needs) and certain criteria (such as physical dimensions, sensing capabilities, and connectivity), but it does not address the ecological design aspects of the selected devices. Similarly, the guidelines presented by Baumel et al. [59] allow for estimating the success of digital health interventions by evaluating digital products as a whole (considering usability and patients’ needs, engagement, stability, and continuous improvement), but do not estimate the economic or environmental costs of the enabling technologies.

In contrast to these works, our contribution addresses central limitations that hinder timely HTA analysis and efficient design within the sustainability domain.

Indeed, our framework emphasizes the core functionalities of smart systems and prototypes, aiming to facilitate not only their cost-effectiveness assessment, but also their sustainability evaluation and design in a pragmatic and integrated manner.

To demonstrate this, we applied our framework to analyze the core functions of smart inhalers designed for COPD patients, resulting in the design of three distinct pilot devices. In the economic context, for the same benefits obtained over a five-year period, we found that smart inhaler prototypes with a high-performance design are less costly than those with a feature-rich design (refer to Table 6). Conversely, in the environmental context, for the same savings obtained over the same period, smart inhaler prototypes with a high-performance design are slightly more costly than those with a lightweight design (refer Table 11).

**Figure 5:**
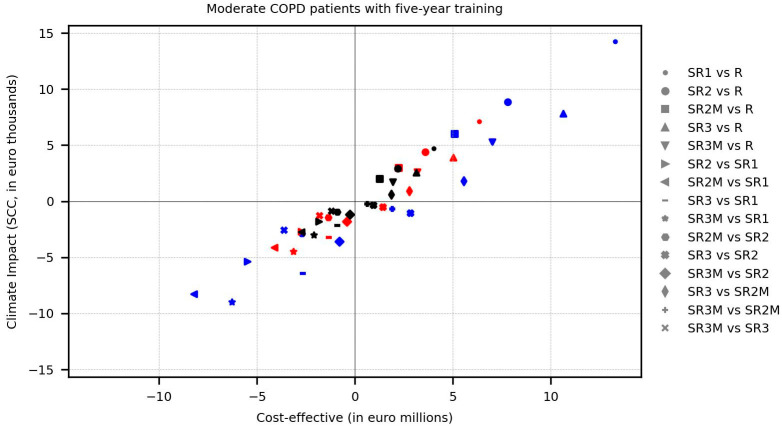
Incremental Cost-Effectiveness Ratios (ICER) of the studied policies under treatment patterns 1, 2 and 3 (blue, red and black marks, respectively). This is a simplified cost-effectiveness analysis, with each mark showing the arithmetic difference between the costs of a “new” policy vs. the costs of a “current” one. It establishes a “which of two alternatives is less costly for at least as much benefit” evaluation strategy [61] (that is, by omitting the estimation of the Quality-Adjusted Life Years (QALYs) values to simplify the analysis).

As such, our framework provides a structured basis not only for identifying critical aspects that guide decision-making in the ecological design of final IoMT devices and systems, but also for shaping sustainable policy interventions. Consider, for example, Figure 5 below, which adapts the sustainable net, cost-effective balance framework proposed by Raymakers, A. J. N., et al. [60] for comparing the economic and ecologic Incremental Cost-Effectiveness Ratio (ICER) of policies studied in this article.

As observed, our estimations show that accounting for the environmental impacts and economic costs of integrated circuits (IC) in electronic modules is of paramount importance. From the environmental perspective, the difference in impact between the use of smart reusable inhalers with a feature-rich design (included in the policy configuration SR1) and the use of reusable inhalers (benchmark policy R) is substantial across all treatment patterns (small circle marks). However, it can be significantly reduced by using simpler microcontrollers in a high-performance design version of smart inhalers (included in policy configuration SR2, big circle marks) and marginally reduced by opting for simpler memory and sensors in the lightweight design version of smart inhalers (included in policy configuration SR3, upward-pointing triangle marks). Moreover, using both design versions exclusively with patients who did not correct their inhalation technique (refer to policy configuration SR2M and SR3M) moderately decreases the gap from reusable inhalers (square and downward-pointing triangle marks), while combined with a policy based exclusively on treatment pattern 3 (black marks), it achieves a markedly greater reduction. However, the above applies only to the policy configuration SR2M within the economic context, as the smart inhalers with a lightweight design used in the policy configuration SR3M unfortunately employ more expensive sensors (albeit slightly more environmentally friendly).

Our framework also enables a first-order cost-effectiveness appreciation, providing rapid insights into whether the introduction of specific IoMT devices into health systems is worthwhile by means of multiple strategies for assessing the associated benefits. Indeed, throughout our analysis, we found that the use of smart inhalers presents more drawbacks than advantages—at least in the context of our assumptions and our tactics for modeling the adjacent benefit. This is critical for simplifying the decision to either adjust our evaluation strategy early or simply abandon the idea of replacing reusable inhalers with smart reusable inhalers in the mid-term (even gradually), as our estimates indicate that the environmental and economic investments required to progressively improve drug delivery through smart inhalers exceed the environmental and economic investments of treating COPD patients with non-smart reusable inhalers.

On the other hand, although our methodological framework was applied to a case study involving a specific disease, it is designed to highlight the fundamental role of information interpretation in a general context, thereby facilitating its systematic use in estimating the benefits of enabling technology in digital health. For example, in the context of cardiovascular diseases, beyond leveraging atypical data sources to monitor and detect cardiac dysfunctions through IoT technology embedded in electrocardiogram equipment [62], our framework would also help emphasize the importance of the frequency of such anomalies as an interpretation of the improvement or deterioration in the patient’s quality of life. Similarly, in the context of neurodegenerative diseases, in addition to passively collecting smartphone sensor data (e.g., accelerometer data) to track the progression of amyotrophic lateral sclerosis [63], our framework would further underscore the relevance of these data at specific time points as a mode of interpretation of the effectiveness of complementary therapies, such as physiotherapy aimed at maintaining mobility and preventing contractures.

Finally, as with any back-of-the-envelope estimation, this research is based on certain assumptions and presents limitations in its results, to which the reader should pay particular attention. Regarding assumptions, it is presumed that clinical outcomes derived from using smart inhalers and from using regular inhalers are, at least, the same. Also, it is assumed that the clinical outcomes when passing from using smart inhalers to using regular ones (policy configuration SR2M and SR3M) are unchanged. On the other hand, the Monte Carlo models assume the normal distribution of drug delivery rates, with a linear improvement of inhalation technique over the years for simplicity. In addition, it is acknowledged that the analysis of aspects related to patients (e.g., acceptance and preference around use or cessation of use of smart inhalers, behavioral regression, etc.) is absent in this study.

Regarding the results, it is recognized that the estimations of environmental costs are based exclusively on global warming in relation to producing smart reusable inhalers, to be coherent with the reference study. Moreover, economic and environmental uncertainties arising throughout the life cycle of smart inhalers (e.g., industrial scalability or waste flow of electronic modules) are not taken into account.

## 5. Conclusions

Smart technologies offer both advantages and drawbacks for digital health. The multitude of contributing factors on either side make the development and implementation of efficient and responsible innovations in the medical field a formidable challenge—particularly in the absence of specialized tools capable of catalyzing the agile and effective deployment of HTA to support early ecological design. In this article, we introduce a novel three-stage HTSA framework designed to address this gap, specifically within the domain of smart technologies and the context of IoMT systems. The framework is grounded in information science theory and two hypotheses, themselves derived from an abstraction of the fundamental purpose of such technologies.

Through the application of our approach to a specific case study involving the deployment of smart inhalers in the treatment of COPD, we found evidence supporting our second hypothesis; specifically, that even a limited set of information can enable the adoption of pragmatic strategies to quantify the costs and intrinsic benefits of smart assisted inhalation medication, while simultaneously facilitating the economic and environmental projection of policies implementing the partial or full use of three types of smart inhaler prototypes in the medium term. Regarding our first hypothesis, we found that the effectiveness of smart inhalers in increasing drug delivery for COPD treatment may be quite limited, while the implementation costs are relatively high (at least based on the specific information interpreted here). However, we also acknowledge the potential of smart inhaler prototypes based on pressure drop sensing with high-performance and lightweight designs, especially when combined with mixed policies.

In this manner, our framework positions itself as a preliminary tool for assessing the potential benefits and risks of smart technologies—potentially even prior to large-scale industrial production. However, it is not intended to replace the empirical rigor of clinical studies, but rather to complement and accelerate them.

Importantly, the case study analyzed in this work focuses solely on the economic and ecological dimensions of HTSA, and was developed through a simulation to estimate the cost and potential benefits of using three types of smart devices before the design of interventional studies. In future research, we aim to address these limitations and explore the transferability of our framework to other medical devices used in the treatment of different conditions, particularly neurodegenerative diseases. Through this process, we also expect to gather clinical evidence that may reinforce or reshape our main hypotheses, and also help address the research question raised in this article.

## Figures and Tables

**Table 1:** Electronic designs of the three versions of the electronic module.

Design Version	MCU *	NFC-EEPROM Memory	Pressure Sensors (2×)	Overall
Feature-rich (A)	STM32L4A6AGI6 [52]	ST25DV04K-IE6C3 [53]	LPS22DF [54]	Best for advanced prototypes and diagnostic functions
High-performance (B)	STM32L051K6U6 [55]	ST25DV04K-IE6C3	LPS22DF	Best for optimized low-power prototypes with high sensing quality
Lightweight (C)	STM32L051K6U6	ST25TN01K [56]	LPS25HB [57]	Best for cost-sensitive and ultra-low-power prototypes with basic performance

* MCU = microcontroller.

**Table 2:** Economic costs and environmental impacts of the electronic module in its three versions.

Design Version	Economic Cost (EUR)	Env. Impact (kg CO_2_-eq)
A	12.32	0.317
B	7.46	0.197
C	9.97	0.174

**Table 3:** Annual costs of smart inhaler prototypes.

Treatment Pattern	Economic Cost (in Millions of EUR) *			Env. Cost (SCC in Thousands of EUR) **		
	A	B	C	A	B	C
1	64.5	59.0	61.8	50.3	44.9	43.9
2	56.5	53.7	55.2	27.8	25.1	24.6
3	53.8	52.0	52.9	20.3	18.5	18.2

* Assuming a production cost of EUR 42.96 per cartridge and EUR 1.87 per reusable inhaler case, according to estimations from the reference study [44]. ** Assuming an environmental impact of 1.035 kg CO_2_-eq per reusable inhaler case [44] with a standard-content cartridge generating approximately 80 g CO_2_-eq [51].

**Table 4:** Average increase in the annual delivery rates of Spiriva for patients that are likely to adopt the proper inhalation technique.

Training Year	Five-Year Training		Four-Year Training	
	Moderate COPD	Severe COPD	Moderate COPD	Severe COPD
2019	0.714%	0.475%	0.000% *	0.000% *
2020	1.517%	1.284%	0.721%	0.478%
2021	2.308%	2.069%	1.520%	1.279%
2022	3.101%	2.880%	2.323%	2.072%
2023	3.929%	3.646%	3.122%	2.873%

* The average delivery rate for 2019 is assumed to zero, as the four-year training process starts from 2020 (consistent with the reference study).

**Table 5:** Annual cost of a reduced-content Spiriva cartridge (expressed in EUR).

Post-Training Year	Five-Year Training		Four-Year Training	
	Moderate COPD	Severe COPD	Moderate COPD	Severe COPD
2024	42.65	42.76	42.96	42.96
2025	42.31	42.41	42.65	42.75
2026	41.97	42.07	42.31	42.41
2027	41.63	41.72	41.96	42.07
2028	41.27	41.39	41.62	41.73

**Table 6:** Average costs of the default policy during the post-training period, under the treatment patterns 1, 2 and 3 (in millions of EUR).

Training Period	COPD Severity	SR1			SR2			SR3		
		Pattern 1	Pattern 2	Pattern 3	Pattern 1	Pattern 2	Pattern 3	Pattern 1	Pattern 2	Pattern 3
Five-year	Moderate	64.14	56.13	53.46	58.65	53.39	51.63	61.48	54.80	52.58
	Severe	64.17	56.17	53.50	58.69	53.43	51.67	61.52	54.84	52.61
Four-year	Moderate	64.32	56.32	53.65	58.84	53.57	51.82	61.67	54.99	52.76
	Severe	64.34	56.34	53.67	58.86	53.59	51.84	61.69	55.01	52.78

**Table 7:** Annual savings of the default policy (in millions of EUR).

Post-Training Year	Five-Year Training		Four-Year Training	
	Moderate COPD	Severe COPD	Moderate COPD	Severe COPD
2024	0.112	0.074	0.000	0.000
2025	0.237	0.200	0.082	0.055
2026	0.361	0.323	0.173	0.147
2027	0.485	0.450	0.265	0.238
2028	0.615	0.569	0.356	0.330

**Table 8:** Average costs and savings of the alternative policy in its SR2M configuration during the post-training period, under the treatment patterns 1, 2 and 3 (in millions of EUR).

Training Period	COPD Severity	Cost			Savings		
		Pattern 1	Pattern 2	Pattern 3	Pattern 1	Pattern 2	Pattern 3
Five-year	Moderate	55.93	52.03	50.73	3.08	1.72	1.27
	Severe	55.98	52.07	50.77	3.03	1.68	1.23
Four-year	Moderate	56.86	52.58	51.16	2.15	1.16	0.83
	Severe	56.87	52.60	51.18	2.15	1.15	0.82

**Table 9:** Average costs and savings of the alternative policy in its SR3M configuration during the post-training period, under the treatment patterns 1, 2 and 3 (in millions of EUR).

Training Period	COPD Severity	Cost			Savings		
		Pattern 1	Pattern 2	Pattern 3	Pattern 1	Pattern 2	Pattern 3
Five-year	Moderate	57.85	52.99	51.37	3.99	2.18	1.57
	Severe	57.90	53.03	51.41	3.95	2.13	1.53
Four-year	Moderate	59.03	53.67	51.88	2.82	1.50	1.06
	Severe	59.03	53.68	51.90	2.82	1.48	1.04

**Table 10:** Annual cost of a reduced-content Spiriva cartridge (expressed in SCC, EUR cents).

Post-Training Year	Five-Year Training		Four-Year Training	
	Moderate COPD	Severe COPD	Moderate COPD	Severe COPD
2024	0.318	0.318	0.320	0.320
2025	0.315	0.316	0.318	0.318
2026	0.313	0.313	0.315	0.316
2027	0.310	0.311	0.313	0.313
2028	0.307	0.308	0.310	0.311

**Table 11:** Average costs of the default policy during the post-training period, under treatment patterns 1, 2 and 3 (expressed in SCC, thousands of EUR).

Training Period	COPD Severity	SR1			SR2			SR3		
		Pattern 1	Pattern 2	Pattern 3	Pattern 1	Pattern 2	Pattern 3	Pattern 1	Pattern 2	Pattern 3
Five-year	Moderate	50.30	27.81	20.32	44.92	25.12	18.52	43.87	24.60	18.17
	Severe	50.31	27.81	20.32	44.92	25.12	18.52	43.88	24.60	18.17
Four-year	Moderate	50.32	27.83	20.33	44.93	25.13	18.53	43.89	24.61	18.19
	Severe	50.32	27.83	20.33	44.94	25.14	18.54	43.89	24.61	18.19

**Table 12:** Annual savings of the default policy (expressed in SCC, thousands of EUR).

Post-Training Year	Five-Year Training		Four-Year Training	
	Moderate COPD	Severe COPD	Moderate COPD	Severe COPD
2024	0.008	0.006	0.000	0.000
2025	0.018	0.015	0.006	0.004
2026	0.027	0.024	0.013	0.011
2027	0.036	0.033	0.020	0.018
2028	0.046	0.042	0.026	0.025

**Table 13:** Average costs and savings of the alternative policy in its SR2M configuration during the post-training period, under treatment patterns 1, 2 and 3 (expressed in SCC, thousands of EUR).

Training Period	COPD Severity	Cost			Savings		
		Pattern 1	Pattern 2	Pattern 3	Pattern 1	Pattern 2	Pattern 3
Five-year	Moderate	42.04	23.68	17.56	2.90	1.47	0.99
	Severe	42.05	23.69	17.57	2.89	1.46	0.98
Four-year	Moderate	42.84	24.09	17.84	2.11	1.06	0.71
	Severe	42.83	24.08	17.83	2.12	1.07	0.71

**Table 14:** Average costs and savings of the alternative policy in its SR3M configuration during the post-training period, under the treatment patterns 1, 2 and 3 (expressed in SCC, thousands of EUR).

Training Period	COPD Severity	Cost			Savings		
		Pattern 1	Pattern 2	Pattern 3	Pattern 1	Pattern 2	Pattern 3
Five-year	Moderate	41.33	23.33	17.33	2.57	1.30	0.87
	Severe	41.34	23.33	17.33	2.56	1.29	0.87
Four-year	Moderate	42.04	23.69	17.57	1.86	0.94	0.63
	Severe	42.03	23.68	17.57	1.87	0.94	0.63

**Figure A1:**
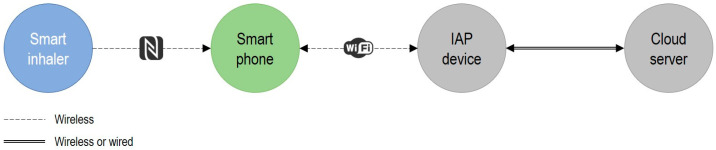
Basic deployment of an IoMT system for inhaler-based COPD treatment. Note: IAP stands for Internet Access Point.

## Data Availability

The data used in this research work are available from the corresponding authors upon requests.

## Supplementary Materials

The following supporting information can be downloaded at: https://www.mdpi.com/article/10.3390/s25133839/s1, Table S1: Reference inspiration flow rate values and slopes obtained from the graphical interpolation of the inhalation profile of a patient with moderate COPD [46].

## Appendix A

The reference study [44] considers 365,000 COPD patients who follow treatment pattern 1 (45%), treatment pattern 2 (45%) or treatment pattern 3 (10%), receiving Spiriva ^®^ (57%), Spiolto ^®^ (42%) or Striverdi ^®^ (1%). To ensure the comparability of the treatment patterns and preserve the previous estimations made for treatment patterns 1 and 2, we increased the original sample size to 492,750 individuals, and adjusted the distribution of the treatment patterns according to Table A1 below.

**Table A1:** Preparation of the original data.

Treatment Pattern	Spiriva (57%)	Spiolto (42%)	Striverdi (1%)
1 (33.3%) **	94,050 *	68,850 *	1350 *
2 (33.3%) **	94,050 *	68,850 *	1350 *
3 (33.3%) **	94,050 **	68,850 **	1350 **
Total	282,150 **	206,550 **	4050 **

* Original sample size estimated from the reference study. ** Modified.

Accordingly, as outlined in the research methodology of this study, our analysis was restricted to the available data related to the treatment of COPD with Spiriva (second column), encompassing 94,050 patients adhering to treatment pattern 1 (involving 1,128,600 inhalers and 1,128,600 cartridges per year), 94,050 patients adhering to treatment pattern 2 (involving 564,300 inhalers and 1,128,600 cartridges per year), and 94,050 patients adhering to treatment pattern 3 (involving 376,200 inhalers and 1,128,600 cartridges per year).

## Appendix B

Table A2, Table A3 and Table A4 present, respectively, the basic designs of the feature-rich (A), high-performance (B) and lightweight (C) versions of the electronic module, along with the economic costs and environmental impacts of their electronic components and assembly processes.

**Table A2:** Basic design and detailed economic cost and global warming impacts of the feature-rich version A of the electronic module.

Component or Process	IPCC * (kg CO_2_-eq)	Cost (EUR) **
STM32L4A6AGI6 x1	1.87 × 10^−1^	6.20
ST25DV04K-IE6C3 x1	2.20 × 10^−2^	0.65
LPS22DF x1	3.73 × 10^−2^	1.69
LPS22DF x1	3.73 × 10^−2^	1.69
PCB *** (Ø 2 cm) x1	3.30 × 10^−2^	0.58
Coin cell battery x1	7.32 × 10^−5^	1.09
Assembly ****	5.11 × 10^−4^	0.42
Total	3.17 × 10^−1^	12.32

* Intergovernmental Panel on Climate Change (IPCC) Life Cycle Impact Assessment (LCIA) methodology (Global Warming Potential with a 100-year horizon (GWP100)). ** Wholesale quoted prices, as of April 2025. *** Printed Circuit Board (PCB) FR4, double-faced, HASL lead-free (environmental impact and costs calculated primarily based on the final area). **** The environmental impact was calculated primarily based on the aggregated footprint areas of the electronic components. The economic cost was calculated primarily based on the number and type of the electronic components and their number of pines.

**Table A3:** Basic design and detailed economic cost and global warming impact of the high-performance version B of the electronic module.

Component or Process	IPCC * (kg CO_2_-eq)	Cost (EUR) **
STM32L051K6U6 x1	6.75 × 10^−2^	1.34
ST25DV04K-IE6C3 x1	2.20 × 10^−2^	0.65
LPS22DF x1	3.73 × 10^−2^	1.69
LPS22DF x1	3.73 × 10^−2^	1.69
PCB *** (Ø 2 cm) x1	3.30 × 10^−2^	0.58
Coin cell battery x1	7.32 × 10^−5^	1.09
Assembly ****	3.16 × 10^−4^	0.42
Total	1.97 × 10^−1^	7.46

* Intergovernmental Panel on Climate Change (IPCC) Life Cycle Impact Assessment (LCIA) methodology (Global Warming Potential with a 100-year horizon (GWP100)). ** Wholesale quoted prices, as of April 2025. *** Printed Circuit Board (PCB) FR4, double-faced, HASL lead-free (environmental impact and costs calculated primarily based on the final area). **** The environmental impact was calculated primarily based on the aggregated footprint areas of the electronic components. The economic cost was calculated primarily based on the number and type of the electronic components and their numbers of pines.

**Table A4:** Basic design and detailed economic cost and global warming impacts of the lightweight version C of the electronic module.

Component or Process	IPCC * (kg CO_2_-eq)	Cost (EUR) **
STM32L051K6U6 x1	6.75 × 10^−2^	1.34
ST25TN01K x1	6.24 × 10^−3^	0.22
LPS25HB x1	3.35 × 10^−2^	3.16
LPS25HB x1	3.35 × 10^−2^	3.16
PCB *** (Ø 2 cm) x1	3.30 × 10^−2^	0.58
Coin cell battery x1	7.32 × 10^−5^	1.09
Assembly ****	3.23 × 10^−4^	0.42
Total	1.74 × 10^−1^	9.97

* Intergovernmental Panel on Climate Change (IPCC) Life Cycle Impact Assessment (LCIA) methodology (Global Warming Potential with a 100-year horizon (GWP100)). ** Wholesale quoted prices, as of April 2025. *** Printed Circuit Board (PCB) FR4, double-faced, HASL lead-free (environmental impact and costs calculated primarily based on the final area). **** The environmental impact was calculated primarily based on the aggregated footprint areas of the electronic components. The economic cost was calculated primarily based on the number and type of the electronic components and their numbers of pines.

All smart inhaler prototypes operate under a very basic IoMT system (Figure A1), where the patient must bring his or her smart inhaler close to his or her mobile phone after the first inhalation and wait for a response, which provides the necessary feedback to improve their technique after a few seconds.
