# Supplementary material for: Towards the Operationalization of Health Technology Sustainability Assessment and the Early Eco Design of the Internet of Medical Things
Source: Sensors (Basel). 2025 Jun 20;25(13):3839. doi: 10.3390/s25133839 (PMC12251846; doi:10.3390/s25133839)
Supplement: Supplementary file 1 [file sensors-25-03839-s001.zip › sensors-3645322-supplementary.pdf]

Towards the Operationalization of Health Technology Sustainability Assessment and the Early Eco Design of the Internet of Medical Things

(Supplementary material 1)

Table S1 below shows the reference inspiration flow rates (second columns) obtained from the graphical interpolation of 18 datapoints of the correspondent inhalation profiles of patients with moderate and severe COPD (presented in previous literature [46]), as well as the calculated slopes (fourth columns), here interpretated as the pressure drop experienced when a smart prototype is used.

Moderate COPD				
**Data point**	**Flow rate (L/min)**	**Time (s)**	**Slope (m)**	**Volume (ml)**
1	0	0,15		
2	68	0,20	1360	28
3	76	0,25	160	60
4	92	0,50	64	350
5	94	0,55	40	78
6	94	0,65	0	157
7	76	1,00	-51	496
8	62	1,20	-70	230
9	48	1,45	-56	229
10	38	1,65	-50	143
11	28	2,00	-29	193
12	22	2,20	-30	83
13	18	2,40	-20	67
14	12	2,80	-15	100
15	10	3,00	-10	37
16	8	3,25	-8	38
17	6	3,55	-7	35
18	4	4,00	-4	38
			**Total**	**2360**

Severe COPD				
**Data point**	**Flow rate (L/min)**	**Time (s)**	**Slope (m)**	**Volume (ml)**
1	0	0,15		
2	54	0,20	1080	23
3	58	0,25	80	47
4	58	0,30	0	48
5	68	0,55	40	263
6	68	0,65	0	113
7	66	0,75	-20	112
8	56	1,00	-40	254
9	47	1,20	-45	172
10	37	1,45	-40	175
11	29	1,70	-32	138
12	21	2,00	-27	125
13	14	2,40	-18	117
14	11	2,70	-10	63
15	8	3,00	-10	48
16	6	3,30	-7	35
17	5	3,65	-3	32
18	4	4,00	-3	26
			**Total**	**1788**

Table S1. Reference inspiration flow rate values and slopes obtained from the graphical interpolation of the inhalation profile of a patient with moderate COPD.

Accordingly, the following illustrates the simulation of the annual inhalation profile of a patient with moderate COPD (simplified to 18 points), who progressively increased their inspiratory volume thanks to the use of a smart prototype, reflecting an improved inhalation technique each year from 2024 to 2028.

2024					
**Flow rate (L/min)**	**Time (s)**	**Slope**	**Error**	**Volume (ml)**	**SD**
0,000000000	0,15	0,000000000	0,000000000		
67,99418587	0,2	1359,970929	0,999978625	28,33091078	0,5
76,20677859	0,25	160,8271143	1,005169465	60,08373519	0,470588235
92,21651434	0,5	64,43302867	1,006766073	350,8818603	0,441176471
94,23312508	0,55	40,42386379	1,010596595	77,68734976	0,411764706
93,88948987	0,65	-0,170015577	0,000000000	156,4824831	0,382352941
76,45763649	1	-50,97036351	0,991101414	496,8457852	0,352941176
62,48309892	1,2	-69,59741756	0,994248822	231,5678924	0,323529412
49,1983173	1,45	-55,17357428	0,985242398	232,6696171	0,294117647
39,4422246	1,65	-49,12592449	0,98251849	147,7342365	0,264705882

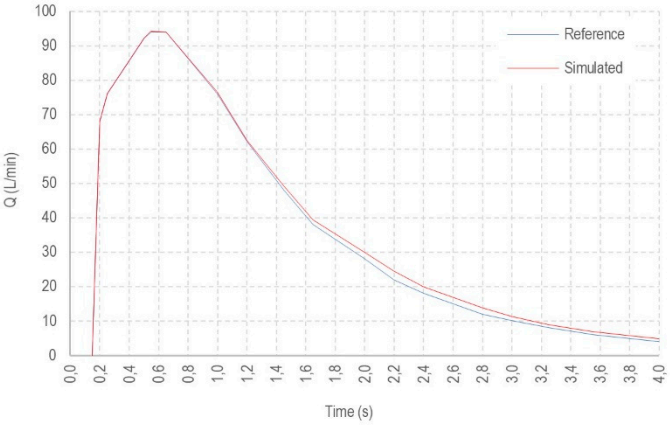

29,90534252	2	-27,61732874	0,966654839	202,2637374	0,235294118
24,42702576	2,2	-28,89680647	0,963226882	90,55394713	0,205882353
19,90291309	2,4	-19,20711954	0,960355977	73,88323142	0,176470588
13,86670146	2,8	-14,33332091	0,955554727	112,5653818	0,147058824
11,19055076	3	-9,603149746	0,960314975	41,76208704	0,117647059
8,846971382	3,25	-7,739393421	0,967424178	41,7448378	0,088235294
6,912530071	3,55	-6,407738008	0,962122824	39,39875363	0,058823529
4,746616426	4	-4,255845893	0,95852385	43,72179936	0,029411765
			**Total**	**2428,177646**	

2025					
**Flow rate (L/min)**	**Time (s)**	**Dif Pressure**	**Error**	**Volume (ml)**	**SD**
0,000000000	0,15	0,000000000	0,000000000		
68,03027494	0,2	1360,151375	1,000132683	28,34594789	0,4
76,27370866	0,25	161,0948346	1,001664646	60,12665983	0,376470588
92,52526703	0,5	65,05053407	1,009583678	351,6645327	0,352941176
94,03971233	0,55	40,07220424	0,991300694	77,73540807	0,329411765
93,69083278	0,65	-0,475641884	2,797637086	156,151388	0,305882353
76,69563639	1	-50,73236361	0,995330622	496,9605351	0,282352941
62,60968814	1,2	-69,49192655	0,998484268	232,1755409	0,258823529
49,42604292	1,45	-55,01652212	0,99715349	233,407773	0,235294118
39,32664661	1,65	-49,19597175	1,001425872	147,9211492	0,211764706
30,28075744	2	-27,42962128	0,993203272	203,0215952	0,188235294
25,07515629	2,2	-28,60220169	0,989804936	92,25985622	0,164705882
20,41654338	2,4	-18,99310693	0,988857641	75,81949945	0,141176471
14,42666782	2,8	-14,13333292	0,986047338	116,1440373	0,117647059
11,71815431	3	-9,427281896	0,981686441	43,57470355	0,094117647
9,546519745	3,25	-7,524147771	0,972188305	44,30140428	0,070588235
7,358879118	3,55	-6,282005882	0,980378079	42,26349716	0,047058824
4,997065464	4	-4,193233634	0,98528794	46,33479218	0,023529412
			**Total**	**2448,20832**	

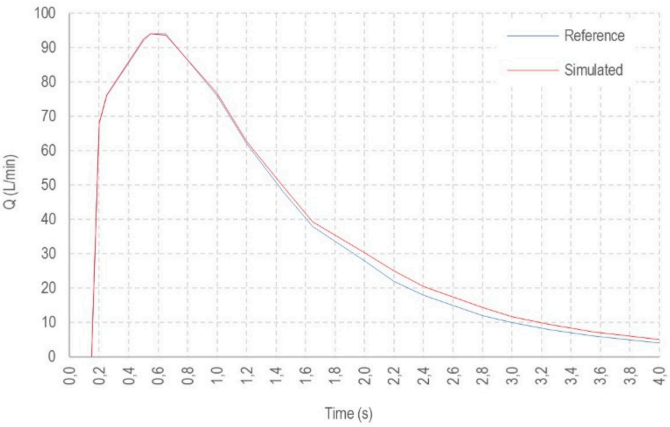

2026					
**Flow rate (L/min)**	**Time (s)**	**Dif Pressure**	**Error**	**Volume (ml)**	**SD**
0,0000000000	0,15	0,0000000000	0,0000000000		
67,96820772	0,2	1359,841039	0,999771837	28,32008655	0,3
76,32907573	0,25	161,3163029	1,00137477	60,12386811	0,282352941
92,64570649	0,5	65,29141299	1,003702951	352,0307963	0,264705882
93,91110258	0,55	39,83836833	0,994164636	77,73200378	0,247058824
93,88256072	0,65	-0,18067582	0,379856834	156,4709345	0,229411765
76,59421357	1	-50,83378643	1,001999174	497,223925	0,211764706
62,28857009	1,2	-69,75952492	1,003850784	231,4713061	0,194117647
49,21142875	1,45	-55,1645319	1,002690279	232,2916643	0,176470588
39,07738497	1,65	-49,34703941	1,003070732	147,1480229	0,158823529
30,09776187	2	-27,52111906	1,003335729	201,760845	0,141176471
24,94437249	2,2	-28,66164887	1,002078413	91,7368906	0,123529412
20,10486904	2,4	-19,12297123	1,006837444	75,08206921	0,105882353
14,02404724	2,8	-14,27712599	1,010174038	113,7630543	0,088235294
11,49808545	3	-9,500638183	1,007781277	42,53688781	0,070588235

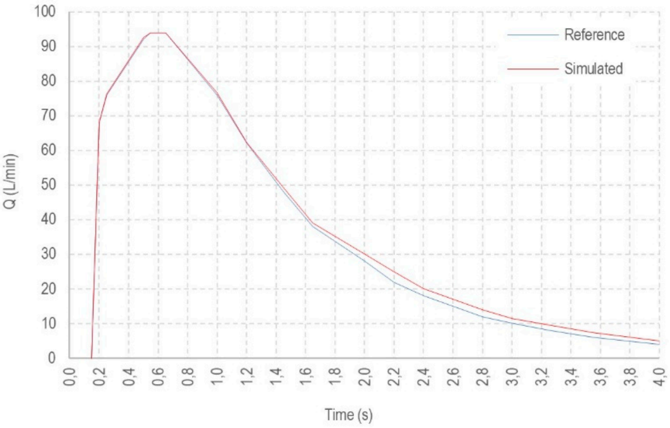

9,508253827	3,25	-7,535921899	1,001564845	43,76320683	0,052941176
7,311009831	3,55	-6,295490188	1,002146497	42,04815915	0,035294118
4,945624625	4	-4,206093844	1,003066896	45,96237921	0,017647059
			**Total**	**2439,466099**	

**2027**					
**Flow rate (L/min)**	**Time (s)**	**Dif Pressure**	**Error**	**Volume (ml)**	**SD**
0,0000000000	0,15	0,0000000000	0,0000000000		
67,9347816	0,2	1359,673908	0,999877095	28,306159	0,2
76,3376094	0,25	161,3504376	1,000211601	60,11349625	0,188235294
92,49144003	0,5	64,98288007	0,995274525	351,7271863	0,176470588
93,70462792	0,55	39,46295985	0,990576711	77,58169498	0,164705882
93,91335769	0,65	-0,133295867	0,737762622	156,5222628	0,152941176
76,78600688	1	-50,64199312	0,996227051	497,8731466	0,141176471
62,54530737	1,2	-69,54557719	0,996933068	232,2188571	0,129411765
49,18525991	1,45	-55,18257937	1,000327157	232,7720152	0,117647059
39,08984201	1,65	-49,33948969	0,999847008	147,1251699	0,105882353
30,14055625	2	-27,49972187	0,999222517	201,9219949	0,094117647
24,96609939	2,2	-28,651773	0,999655433	91,84442607	0,082352941
19,99416699	2,4	-19,16909709	1,002412065	74,93377731	0,070588235
14,04272174	2,8	-14,27045652	0,999532857	113,4562958	0,058823529
11,43783659	3	-9,520721138	1,002113853	42,46759721	0,047058824
9,426808032	3,25	-7,560982144	1,003325438	43,46800962	0,035294118
7,219784175	3,55	-6,321187556	1,004081869	41,61648052	0,023529412
4,83267148	4	-4,23433213	1,006713661	45,19670871	0,011764706
			**Total**	**2439,145278**	

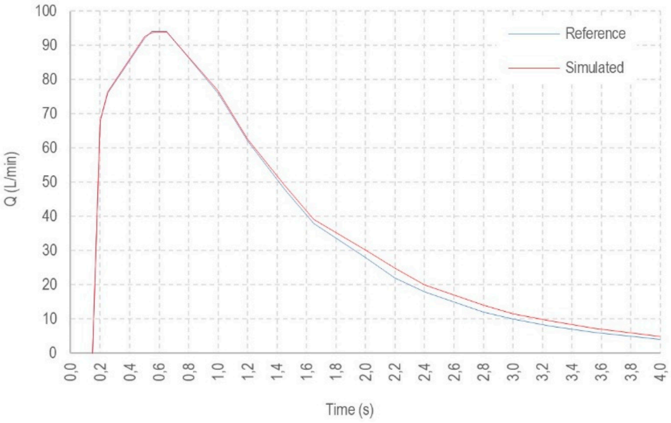

**2028**					
**Flow rate (L/min)**	**Time (s)**	**Dif Pressure**	**Error**	**Volume (ml)**	**SD**
0,0000000000	0,15	0,0000000000	0,0000000000		
67,91929474	0,2	1359,596474	0,999943049	28,29970614	0,1
76,34581934	0,25	161,3832774	1,000203531	60,1104642	0,094117647
92,50120128	0,5	65,00240255	1,000300425	351,7646263	0,088235294
93,77323888	0,55	39,58770706	1,003161121	77,61435007	0,082352941
93,88414556	0,65	-0,178237598	1,337157723	156,4735759	0,076470588
76,66079588	1	-50,76720412	1,002472474	497,4227459	0,070588235
62,29121294	1,2	-69,75732255	1,003044699	231,5866814	0,064705882
48,99839896	1,45	-55,31144899	1,002335332	231,8533581	0,058823529
38,98128385	1,65	-49,40528251	1,001333472	146,6328047	0,052941176
29,90076415	2	-27,61961792	1,0043599	200,9059733	0,047058824
24,75881186	2,2	-28,74599461	1,003288509	91,09929335	0,041176471
19,85339017	2,4	-19,22775409	1,003059978	74,35367006	0,035294118
13,8435497	2,8	-14,34158939	1,004984625	112,3231329	0,029411765
11,20171157	3	-9,599429476	1,008267056	41,74210212	0,023529412
9,226299955	3,25	-7,622676937	1,008159627	42,55835734	0,017647059
7,079029848	3,55	-6,360836663	1,006272414	40,76332451	0,011764706
4,734849283	4	-4,258787679	1,005775539	44,30204674	0,005882353
			**Total**	**2429,806213**	

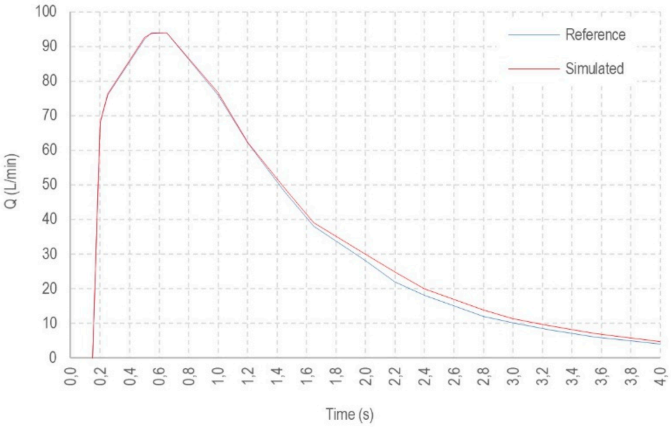

We adopt a very conservative approach. The initial variation of the first slope in the first year (2024) corresponds to a "high" value of the standard deviation (0.5), which is progressively decreased by steps of 0.1 in the next years only if the obtained value for $inhV_{sim}$ overcomes the reference value $inhV$. The error represents the ratio between the simulated value of the slope and the reference value at each data point. The same rationale is applied to patients with severe COPD.

Disclaimer/Publisher's Note: The statements, opinions and data contained in all publications are solely those of the individual author(s) and contributor(s) and not of MDPI and/or the editor(s). MDPI and/or the editor(s) disclaim responsibility for any injury to people or property resulting from any ideas, methods, instructions or products referred to in the content.
